# The Effects of Lidocaine Patches on Post‐Radiofrequency Ablation Pain in Patients With Hepatocellular Carcinoma

**DOI:** 10.1002/cam4.71581

**Published:** 2026-02-15

**Authors:** Chen‐Ju Chen, Wei‐Ying Chen, Chung‐Ying Lin, Yen‐Cheng Chiu, Ying‐Ju Chang

**Affiliations:** ^1^ Department of Nursing National Cheng Kung University Hospital, College of Medicine, National Cheng Kung University Tainan Taiwan; ^2^ Department of Internal Medicine National Cheng Kung University Hospital, College of Medicine, National Cheng Kung University Tainan Taiwan; ^3^ Institute of Allied Health Sciences, College of Medicine, National Cheng Kung University Tainan Taiwan; ^4^ School of Nursing, College of Nursing Kaohsiung Medical University Kaohsiung Taiwan; ^5^ Biostatistics Consulting Center National Cheng Kung University Hospital, College of Medicine, National Cheng Kung University Tainan Taiwan; ^6^ Department of Nursing, Institute of Allied Health Sciences College of Medicine, National Cheng Kung University Tainan Taiwan

**Keywords:** lidocaine patch, post‐radiofrequency ablation pain, the equivalent cumulative dose of residual morphine

## Abstract

**Background and Aim:**

Radiofrequency ablation (RFA) is a standard treatment for patients with early‐stage hepatocellular carcinoma (HCC). However, analgesics are often insufficient in mitigating post‐RFA pain. The lidocaine patch is being investigated as an alternative for acute pain relief, though it has not yet been used for post‐RFA pain. This study aims to evaluate the effect of lidocaine patches on post‐RFA pain for patients with HCC.

**Methods:**

Patients undergoing RFA treatment were randomly assigned to an intervention group, receiving lidocaine patches (LP group), or a control group, receiving placebo patches (PP group). The primary endpoint was the Visual Analog Scale (VAS) pain score. The secondary endpoint was rescue analgesic use, quantified as the equivalent cumulative dose of residual morphine. Both were assessed concurrently every 4 h on the first day post‐RFA.

**Results:**

A total of 86 patients in the LP group and 68 patients in the PP group completed the study. Pain scores did not differ between the two groups at any time point. However, the equivalent cumulative dose of residual morphine was lower in the LP group compared to the PP group, with statistically significant differences at time points T4–T6 (*p* = 0.032, *p* = 0.018, and *p* = 0.032, respectively). After adjusting for confounding factors, multiple linear regression analysis yielded similar results.

**Conclusions:**

VAS pain scores, the primary endpoint, were not significantly different between the two groups. However, the objective equivalent cumulative dose of residual morphine was significantly reduced, implying that the lidocaine patch lowered residual systemic opioid exposure. We conclude that the lidocaine patch may be useful to reduce opioid use post‐RFA.

**Trial Registration:**

ClinicalTrials.gov identifier: NCT05732181

## Graphical abstract

The randomized controlled trial in hepatocellular carcinoma patients following radiofrequency ablation, showing that lidocaine patches reduced cumulative residual morphine dose without differences in pain scores.

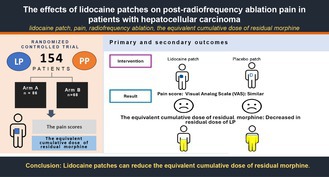

## 1 Introduction

Hepatocellular carcinoma (HCC) is one of the most lethal cancers affecting the digestive tract, ranking among the top five [1]. Additionally, it is the third leading cause of cancer‐related deaths worldwide [2]. For patients in the very early or early stages of HCC where surgery is not recommended, local ablation is a common standard treatment [3, 4]. Percutaneous radiofrequency ablation (RFA) is the primary method of local ablation, typically performed percutaneously. Using real‐time ultrasound guidance, a needle is inserted to coagulate tumor cells through heat conduction, resulting in tumor necrosis [2, 5].

Subsequent to the RFA procedure, 11%–38.4% of patients may experience mild to moderate pain beneath the puncture site within 24–48 h, with the pain peaking in the first 24 h [5, 6, 7]. Clinicians often resort to systemic drugs such as morphine or nonsteroidal anti‐inflammatory drugs (NSAIDs) to manage pain. However, these medications are associated with well‐known side effects, including gastrointestinal discomfort, nausea, vomiting, dizziness, gastrointestinal bleeding, and liver damage [8]. Currently, the lidocaine patch is considered an alternative to opioid pain medication in multimodal treatment plans for acute or chronic pain [9, 10].

The lidocaine patch, known as Lidoderm (Endo Pharmaceuticals Inc., Chadds Ford, PA), is a topical, noninvasive hydrogel pain‐relieving patch that delivers 5% lidocaine through the skin for pain relief. It exerts its effect locally by blocking sodium channels in the nerves of the skin, providing a numbing effect and reducing pain signals [11]. This patch is administered topically and generally safe with relatively minor side effects. Only 3% of lidocaine skin patch drugs enter the systemic circulation [12], reducing the risk of drug interactions and systemic side effects or overdose [13].

The lidocaine patch has demonstrated efficacy in alleviating pain associated with various surgical procedures, including skin abscess surgery, surgical wound pain following cesarean section, and laparoscopic surgery [14, 15, 16]. However, it remains unclear whether the lidocaine patch exerts a comparable effect in mitigating pain among patients with HCC after RFA procedures. Furthermore, previous studies on pain control only focused on the equivalent consumption of cumulative opioid without factoring in the effect of residual rescue analgesics. In addition, pain level was assessed at the end of these studies, in which recall bias might arise [14, 15, 16]. Consequently, this study aims to evaluate the impact of lidocaine patches on pain levels, rescue analgesic use, and residual analgesic concentration in HCC patients post‐RFA treatment.

## 2 Methods

### 2.1 Study Design

This study was conducted with a block‐randomized clinical trial from December 2016 to December 2017 in a medical center of southern Taiwan. The study adhered to the reporting guidelines of the Consolidated Standards of Reporting Trials (CONSORT) [17]. This study obtained approval from the Institutional Review Board of the National Cheng Kung University Hospital (IRB: B‐ER‐106‐060).

All study participants were asked to provide written informed consent after receiving comprehensive oral information from the researcher regarding the study's objectives and procedures. Upon agreeing to participate and signing the informed consent, eligible participants were assigned to either the lidocaine patches (LP) group or the placebo patches (PP) group using permuted block randomization.

### 2.2 Participants and Setting

The eligible participants were 180 patients with HCC who underwent RFA. Participants' inclusion criteria were (1) 18 years or older, (2) with or without liver cirrhosis, but the Child‐Pugh score was less than 8, and (3) able to verbally express their pain. Exclusions were (1) allergic to lidocaine, (2) receiving other treatment except RFA, and (3) having skin diseases or wounds in the abdomen that cannot be patched.

### 2.3 Randomization

A permuted block randomization with a block size of 4 was executed by Random Allocation Software 2.0. The randomization and allocation procedure were conducted independently by a nursing student who was not directly involved in the study. The allocation sequence was managed in a concealed envelope by a third person with no knowledge of the details of the study.

The LP group received lidocaine patches with the plastic covering removed, while the PP group received placebo patches with the covering intact. The patches in both groups were then covered by elastic adhesive tape to ensure that the appearance of both groups appeared indistinguishable. Consequently, participants remained unaware of their group assignment. Pain levels were assessed by primary nurses, who were unaware of the participants' assignment to the LP or PP group.

The sample size was calculated using the G*POWER software for the required participants [18]. Based on a study by de Queiroz et al. [15], we have chosen an effect size of 0.5, a power of 85%, and a significance level of 0.05 for a two‐sided independent *t*‐test. Therefore, 66 participants for each group would be necessary. Considering an attrition rate of 25%, the final sample size for the present study was set at 90 participants per group, for a total of 180 participants.

### 2.4 Intervention

#### 2.4.1 Patch Application and Pain Assessment

Following the RFA procedure, the primary investigator promptly applied the commercially available 5% lidocaine patches (Endo Pharmaceuticals Inc., Chadds Ford, PA) to the LP and PP groups, with the plastic covering removed for the LP group and left on for the PP group. Then, the patches were fixed in place with the same elastic adhesive tape so that the nurses who assessed the outcome pain score were blinded to the two groups. In both groups, patches were placed over the abdominal puncture site and removed after 24 h. Pain levels in both groups were assessed at 4‐h intervals after the RFA procedure for the initial 24 h by primary nurses.

#### 2.4.2 Pain Management

The assessing nurse evaluated the patient's pain condition after the RFA procedure. If a patient indicated intolerable pain or reported a pain score that exceeded 5 points on a scale of 0–10, the nurse inquired about the need for analgesics. Then, all medical care and pain management decisions were determined by attending physicians who were not involved in the research. In this study, we did not alter the attending physicians' decisions regarding rescue medications in real‐world clinical practice. Furthermore, no standardization of analgesics is practiced.

### 2.5 Variables and Data Capture

#### 2.5.1 Primary Outcome: Assessment of Pain Level Differences Between the Two Groups at Various Time Points

The Visual Analogue Scale (VAS) was used for the assessment of pain levels. VAS has demonstrated satisfactory reliability and validity in measuring both chronic and post‐surgery acute pain [19, 20, 21]. Pain scores in VAS range from 0 to 10, with higher scores indicating a higher perceived pain level in this study. Pain levels were consistently measured by the nurse immediately after the RFA procedure for a duration of one day, indicated as baseline (T0), 2 h later (T1), and subsequently every 4 h (T2–T6).

#### 2.5.2 Secondary Outcomes: Rescue Analgesics Information

Patients with severe pain were generally managed with intravenous analgesics, while those with milder pain received oral analgesics. Accordingly, data on analgesic usage—including oral and intravenous doses—were extracted from medical records. In this study we also focused on the equivalent cumulative dose of residual morphine on T2–T6 time points to determine its influence on the VAS score. From a pharmacokinetic perspective, the elimination half‐life of morphine (administered either orally or intravenously) is approximately 2 h, whereas for tramadol, it ranges from 5 to 7 h [22]. In this study, we set the half‐life of morphine at 2 h and that of tramadol at 6 h. Given the relatively low analgesic potency of acetaminophen compared to opioids or weak opioid drugs, it was not considered in our analysis. Additionally, due to potential adverse effects in patients with liver disease, NSAIDs were rarely used for pain management in our routine clinical practice. Finally, because morphine served as the standard analgesic in the study, the dosage of tramadol was converted to equivalent morphine. For example, 100 mg of tramadol was considered equivalent to 10 mg of morphine [23].

Residual presence was assessed according to the half‐life, utilizing an online half‐life calculator (https://miniwebtool.com/zh‐tw/half‐life‐calculator/?n1=&n2=10&n3=8&n4=6) for T2–T6. Afterwards, we summed up each rescue analgesic to calculate the equivalent cumulative dose of residual morphine at time points of T2–T6. Because nearly no patients received rescue analgesics at T0, there was no equivalent cumulative dose of residual morphine at T1.

### 2.6 Statistical Analyses

#### 2.6.1 Descriptive Data

Data were analyzed using IBM/SPSS version 22.0 software. Before performing data analysis, the Kolmogorov–Smirnov test was applied to assess normal distribution. Descriptive statistics were used to summarize the demographic and disease characteristics of the study participants. Differences in demographics and disease characteristics between the two groups were assessed using the chi‐square test for categorical variables and the independent *t*‐test for continuous variables.

#### 2.6.2 Primary Analysis: Pain Level Analyzed by Generalized Estimating Equations (GEE)

The impact of the lidocaine patch on pain level was examined using GEE. Specifically, the interaction effects of the group (LP group compared to PP group) with time (post‐initial reference, T0) on pain levels were estimated to determine whether the LP group demonstrated a reduction in pain levels over time (T1–T6) compared to the PP group. In this study, a significance level of *p* < 0.05 was established for two‐tailed tests. The chi‐square test was used to compare rescue analgesics between groups, with a significance level set at *p* < 0.05. The analysis was performed using a Per‐Protocol approach, excluding participants who violated the protocols of this study.

#### 2.6.3 Secondary Analysis: The Equivalent Cumulative Dose of Residual Morphine Analyzed by Multiple Linear Regression

Further analyses included a comparison of the equivalent cumulative dose of residual morphine between the two groups. We used multiple linear regression to adjust for possible confounding factors.

## 3 Results

**FIGURE 1:**
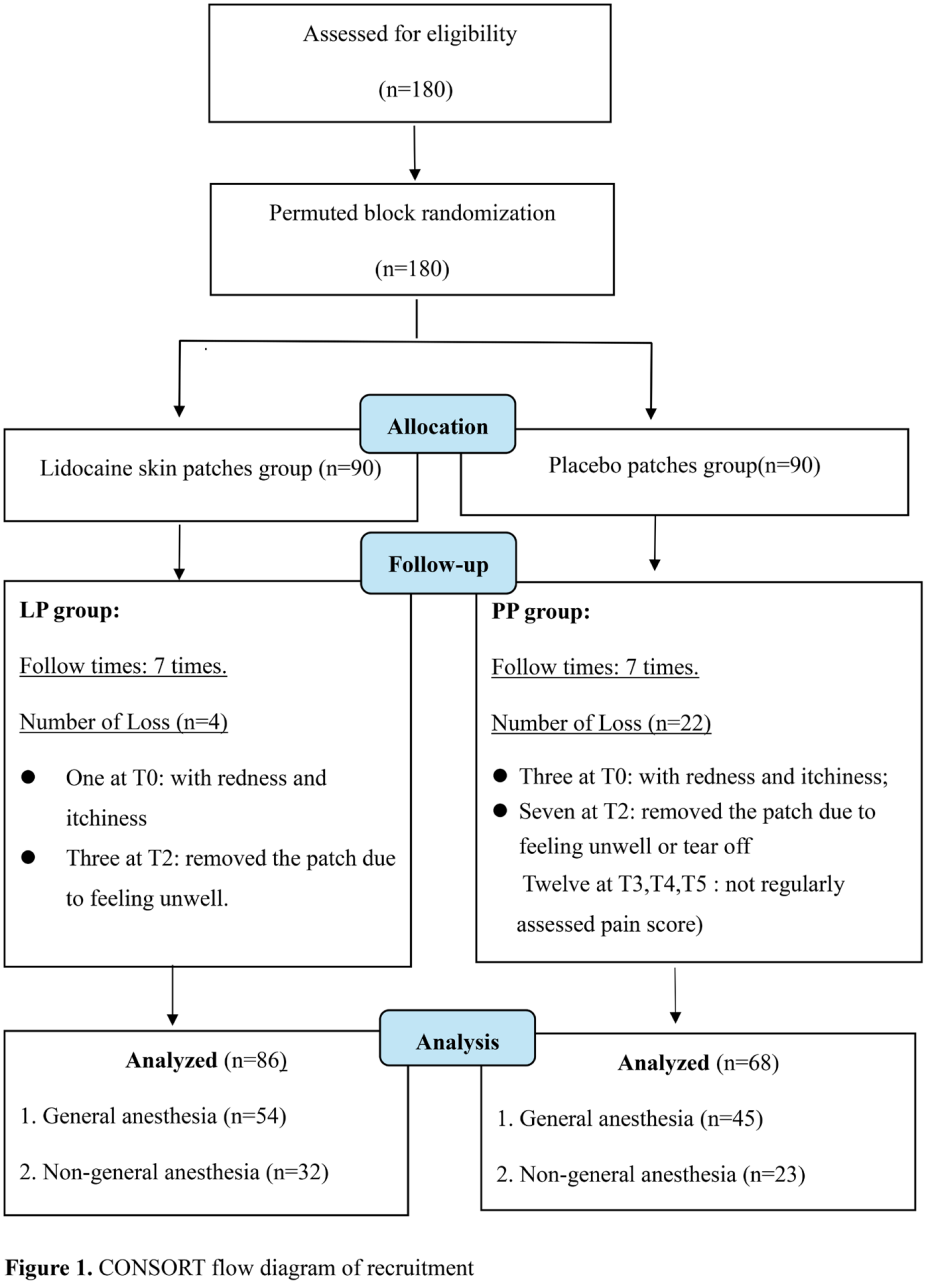
CONSORT flow diagram of recruitment.

Initially, 180 potential participants were assessed for eligibility. During the follow‐up process, four participants in the LP group and 22 participants in the PP group requested the termination of their participation, leading to the decision not to use their data for analyses. Eventually, data from 86 patients in the LP group and 68 patients in the PP group were included in the analysis. Overall, 85.5% (154/180) of the participants successfully completed the study (Figure 1).

### 3.1 Descriptive Data

The mean age (SD) of all participants was 71 (9.66) years with 61% of men. Among them, 60.4% belonged to Barcelona Clinic Liver Cancer (BCLC) staging classification A and 77.9% had small HCC (less than 3 cm). Most RFA procedures were performed under general anesthesia (64.3%). Importantly, no significant differences were observed in the demographic and clinical characteristics between the two groups (Table 1).

**TABLE 1:** Demographic baseline characteristics of the patients.

	Total (*n* = 154)	LP group (*n* = 86)	PP group (*n* = 68)	*χ* ^2^/*t*	*p*
Age: mean (SD)	71.8 (9.7)	71.4 (9.4)	72.2 (1.0)	0.516	0.606
Sex (*n*, %)					
Male	94 (61)	51 (59.3)	43 (63.2)	0.247	0.619
Female	60 (39)	35 (40.7)	25 (36.8)		
Tumor size (*n*, %)					
< 3 cm	120 (77.9)	64 (74.4)	56 (82.4)	1.910	0.385
3–5 cm	33 (21.4)	21 (24.4)	12 (17.6)		
> 5 cm	1 (0.6)	1 (1.2)	0 (0.0)		
BCLC stage (*n*, %)					
A	93 (60.4)	49 (57.0)	44 (64.7)	1.587	0.452
B	60 (39)	36 (41.9)	24 (35.3)		
C	1 (0.6)	1 (1.2)	0 (0.0)		
Cause of hepatitis (*n*, %)					
Unknown	20 (13.0)	11 (12.8)	9 (13.2)	1.983	0.576
B	59 (38.3)	33 (38.4)	26 (38.2)		
C	69 (44.8)	37 (43.0)	32 (47.1)		
Alcohol	6 (3.9)	5 (5.7)	1 (1.5)		
Tumor number (*n*, %)					
One	105 (68.2)	60 (69.0)	45 (66.2)	2.092	0.351
Two	41 (26.6)	20 (23.3)	21 (30.9)		
≥ Three	8 (5.2)	6 (7.0)	2 (2.9)		
Cirrhosis status (*n*, %)					
No	44 (28.6)	25 (29.1)	19 (27.9)	0.079	0.961
C‐P‐T class A	91 (59.1)	50 (58.1)	41 (60.3)		
C‐P‐T class B	19 (12.3)	11 (12.8)	8 (11.8)		
Ascites (*n*, %)					
No	132 (85.7)	73 (84.9)	59 (86.8)	0.110	0.740
Yes	22 (14.3)	13 (15.1)	9 (13.2)		
Anesthesia method for RFA (*n*, %)					
Local	55 (35.7)	33 (37.2)	23 (33.8)	0.190	0.663
General	99 (64.3)	54 (62.8)	45 (66.2)		

Abbreviations: BCLC stage, Barcelona Clinic Liver Cancer (BCLC) staging classification; C‐P‐T, Child‐Pugh‐Turcotte; LP, lidocaine patches; PP, placebo patches; RFA, radiofrequency ablation procedures.

### 3.2 Primary Result: Comparison of Pain Score Between LP and PP Groups

The pain level results indicated no significant difference between the LP and PP groups at any time point after RFA (Table 2). Further analysis using GEE revealed a significant interaction between the groups and the time points (*p* = 0.002), even after accounting for potential confounders such as age, sex, general anesthesia, and the use of intravenous and oral analgesics. Throughout all time points, the pain level for both groups showed a gradual decrease. Importantly, no significant interaction was observed between the groups and time (*p* = 0.851). Furthermore, at T1–T6, the pain level in both groups was similar (Table 3).

**TABLE 2:** Differences in pain scores between the lidocaine patches group and the placebo patches group at different time points.

Time point	LP group (*n* = 86)	PP group (*n* = 68)	*t*	*p*
	Mean ± SD	Mean ± SD		
T0 (Post initial)	2.42 ± 3.05 (*n* = 86)	2.59 ± 2.84 (*n* = 68)	0.357	0.724
T1 (Post 2 h)	1.99 ± 2.27 (*n* = 85)	2.49 ± 2.55 (*n* = 68)	1.276	0.204
T2 (Post 6 h)	1.48 ± 1.59 (*n* = 83)	1.54 ± 1.71 (*n* = 67)	0.204	0.838
T3 (Post 10 h)	1.23 ± 1.79 (*n* = 61)	1.05 ± 1.60 (*n* = 59)	−0.576	0.566
T4 (Post 14 h)	1.77 ± 2.62 (*n* = 31)	1.06 ± 1.53 (*n* = 36)	−1.344	0.168
T5 (Post 18 h)	1.27 ± 1.79 (*n* = 60)	0.89 ± 1.67 (*n* = 54)	−1.165	0.248
T6 (Post 24 h)	1.07 ± 1.34 (*n* = 58)	1.33 ± 1.80 (*n* = 49)	0.849	0.398

Abbreviations: LP, lidocaine patches; PP, placebo patches.

**TABLE 3:** Generalized estimation equation results demonstrate the effects of the lidocaine patch after adjusting for confounding variables.

	Coefficient (SE)	95% CI	*p*
Intercept	2.525 (0.826)	(0.906, 4.144)	0.002*
Group (Ref: control)	0.088 (0.472)	(−0.836, 1.012)	0.851
Time (Ref, T0: post initial)			
T1	0.036 (0.320)	(−0.592, 0.664)	0.911
T2	−0.858 (0.336)	(−1.516, −0.201)	0.0110*
T3	−1.335 (0.368)	(−2.056, −0.613)	< 0.001*
T4	−1.566 (0.359)	(−2.270, −0.861)	< 0.001*
T5	−1.469 (0.414)	(−2.280, −0.657)	< 0.001*
T6	−1.424 (0.447)	(−2.300, −0.549)	0.001*
Time × Group (Ref: control group at baseline)			
T1 for LP	−0.364 (0.446)	(−1.237, 0.510)	0.415
T2 for LP	0.071 (0.495)	(−0.899, 1.041)	0.886
T3 for LP	0.332 (0.571)	(−0.786, 1.450)	0.560
T4 for LP	0.839 (0.680)	(−0.494, 2.171)	0.217
T5 for LP	0.354 (0.586)	(−0.796, 1.503)	0.547
T6 for LP	−0.800 (0.602)	(−1.258, 1.099)	0.894
Age	−0.014 (0.011)	(−0.035, 0.006)	0.167
Sex (Ref: female)	0.226 (0.218)	(−0.202, 0.654)	0.851
GA for RFA procedure (Ref: non‐GA)	0.073 (0.206)	(−0.329, 0476)	0.721
Intravenous analgesia (Ref: non‐intravenous analgesia)	0.237 (1.554)	(1.554, 2.482)	< 0.001*
Oral analgesia (Ref: non‐oral analgesia)	1.577 (0.315)	(0.960, 2.193)	< 0.001*

Abbreviations: CI, confidence interval; GA, general anesthesia; LP, lidocaine patches group; RFA, radiofrequency ablation; SE, standard error.

* *p* < 0.05.

### 3.3 Secondary Outcomes: Comparison of Rescue Analgesics Consumption and the Equivalent Cumulative Dose of Residual Morphine Between Participants With and Without Lidocaine Patch

In Table 4, of the total participants, 61 (40%) used rescue analgesics. A significant difference was observed in the use of oral analgesics between the LP and PP groups, with fewer participants in the LP group resorting to oral analgesics compared to the PP group (*p* = 0.047). In addition, we conducted a calculation of the Number Needed to Treat (NNT), revealing that the application of the lidocaine patch would result in a reduction of oral analgesic use in one out of every seven treated individuals. However, no significant difference was found in the consumption of intravenous analgesics between the two groups (*p* = 0.934).

**TABLE 4:** Comparison of rescue analgesic use between two groups.

Variable	LP group (*n* = 86)	PP group (*n* = 68)	*χ* ^2^	*p*
By orally				
Yes, *n* (%)	8 (5.3)	14 (20.6)	3.950	0.047*
No, *n* (%)	78 (94.7)	54 (79.4)		
Intravenously injection				
Yes, *n* (%)	22 (25.6)	17 (25.0)	0.007	0.934
No, *n* (%)	64 (74.4)	51 (75.0)		

Abbreviations: LP, lidocaine patches; PP, placebo patches.

* *p* < 0.05.

The equivalent cumulative dose of residual morphine in the LP group consistently demonstrated a lower amount compared to the PP group at each measurement point. Statistically significant differences were observed at T4, T5, and T6 time points (*p* = 0.032, *p* = 0.018, and *p* = 0.032, respectively). The observed reduction in analgesic consumption in the LP group, in contrast to the PP group, was further corroborated by the independent *t*‐test, as presented in Table 5.

**TABLE 5:** Comparison of equivalent cumulative dose of residual morphine between two groups at various time points.

Time point	LP group (*n* = 86)	PP group (*n* = 68)	*t*	*p*
	Mean ± SD	Mean ± SD		
T2	7.95 ± 17.62	9.74 ± 18.78	0.609	0.544
T3	5.07 ± 10.68	9.37 ± 17.99	1.848	0.067
T4	2.5 3 ± 5.34	6.16 ± 14.31	2.168	0.032*
T5	0.45 ± 2.24	2.69 ± 7.40	2.659	0.018*
T6	0.09 ± 0.81	0.78 ± 2.82	2.170	0.032*

Abbreviations: LP, lidocaine patches; PP, placebo patches.

* *p* < 0.05.

We further used multiple linear regression to compare the equivalent cumulative dose of residual morphine in Table 6. The analysis revealed that all independent variables could account for the variance in the equivalent cumulative dose of residual morphine at each respective time point: 8.5%, 25.2%, 25.2%, 25.3%, and 23.3%. That is, *R* ^2^ ranges from 8.5% to about 25% across 5 time points. What is worth noticing is that the statistically significant difference between the groups was observed in analgesic residual at T4, T5, and T6 (*p* = 0.026, *p* = 0.011, and *p* = 0.033, respectively).

**TABLE 6:** Linear regression analyses in explaining the equivalent cumulative dose of residual morphine in the two groups.

Time point	*B* (standard error)/*p*				
	T2	T3	T4	T5	T6
Age	0.029 (0.151)/0.851	−0.015 (0.126)/0.906	0.007 (0.091)/−0.172	0.001 (0.046)/0.982	0.004 (0.017)/0.810
Sex (Ref: female)	−0.319 (3.098)/0.918	0.427 (2.508)/0.865	−1.583 (1.806)/0.382	−0.446 (0.917)/0.627	−0.296 (0.347)/0.395
Group (Ref: placebo patches)	−2.321 (2.950)/0.433	−4.485 (2.388)/0.062	−3.862 (1.719)/0.026*	−2.257 (0.873)/0.011*	−0.713 (0.330)/0.033*
GA for RFA (Ref: non‐GA)	9.271 (18.184)/0.611	9.680 (14.723)/0.512	4.786 (10.599)/0.652	2.192 (5.382)/0.684	0.466 (2.037)/0.820
BCLC stage B (Ref: stage A)	−0.748 (3.263)/0.819	−1.7872 (0.642)/0.500	−0.841 (1.902)/0.659	−0.285 (0.966)/0.769	0.034 (0.366)/0.925
BCLC stage C (Ref: stage A)	−9.991 (18.943)/0.599	−4.049 (15.337)/0.792	−6.227 (11.042)/0.574	−0.865 (5.607)/0.878	−0.551 (2.122)/0.796
C‐P‐T Class A (Ref: no cirrhosis)	0.248 (3.390)/0.942	−1.309 (2.744)/0.634	−1.914 (1.976)/0.334	−1.513 (1.003)/0.134	−0.577 (0.380)/0.131
C‐P‐T Class B (Ref: no cirrhosis)	−7.793 (5.092)/0.128	−6.765 (4.123)/0.103	−1.848 (2.968)/0.535	−0.853 (1.507)/0.572	−0.068 (0.570)/0.905
*F* (*p*‐value)	1.333 (0.218)	0.969 (0.473)	0.970 (0.472)	0.977 (0.466)	0.820 (0.610)
*R* ^2^ (Adjusted *R* ^2^)	0.085 (0.021)	0.252 (0.063)	0.252 (0.064)	0.253 (0.064)	0.233 (0.054)
VIF	1.024–1.347	1.024–1.347	1.024–1.347	1.024–1.347	

Abbreviations: BCLC, Barcelona Clinic Liver Cancer staging classification; C‐P‐T, Child‐Pugh‐Turcotte; GA, general anesthesia; RFA, radio‐frequency ablation; VIF, variance inflation factor.

* *p* < 0.05.

### 3.4 Side Effects

The treatment was well tolerated, with only 4 treatment‐emergent adverse events reported. These were localized to the application site and classified as mild, consisting of irritation (*n* = 3) and erythema (*n* = 1). No other systemic adverse events, such as headache, dizziness, or nausea, occurred during the study period. In the control group, a total of 10 adverse events occurred, including erythema (*n* = 3) and irritation (*n* = 7). All were mild and localized, with no systemic adverse events reported.

## 4 Discussion

We used subcutaneous infiltration of a local analgesic to investigate its impact on pain relief following patients receiving RFA treatment. Contrary to the results of previous research [11, 15], our study revealed that the LP group, compared to the PP group, did not produce a statistically significant effect on pain relief. However, our findings indicated a reduction in the rescue analgesic use and a decrease in the equivalent cumulative dose of residual morphine in the LP group.

Compared with the previous studies revealing significant effects of analgesics on pain relief, this study shows insignificant effects for the following reasons. Firstly, not restricting the dose and type of rescue analgesics contributed to the observed results. Secondly, the prospective pain assessment was conducted every 4 h, which minimizes recall bias. Thirdly, unlike cutting or superficial skin pain in other studies [11, 15, 16], the pain related to RFA in our study is more complex, resulting not only from puncture injury but also from heat ablation of the tract. Finally, the anatomic layers of the puncture tract caused by the RFA procedure went through from the skin layers like the epidermis, the dermis, the subcutaneous tissue to the deeper layers like the muscle, the parietal peritoneum, the visceral peritoneum, and the liver. Our study suggests that while the lidocaine patch may address superficial discomfort, it appeared insufficient for mitigating pain originating from these deeper anatomical layers.

To better understand whether the two groups exhibited variations in the consumption of rescue analgesics for pain relief, the use of analgesics was examined as a secondary outcome in our study. The results revealed a significantly lower proportion of patients in the LP group required oral analgesics for post‐RFA pain relief, indicating a decreased need for oral analgesics due to lower pain scores. Our findings align with previous reports [15, 16, 24].

Moreover, we observed fewer residual analgesic concentrations in the LP group, indicative of a lower pain threshold, suggesting superior pain control. Therefore, we conclude that the lidocaine patch could contribute to a reduction in the use of rescue oral opioids and residual analgesic concentrations, thereby maintaining effective pain relief for patients undergoing RFA. Our study also supports the previous studies [16, 25] that lidocaine patches were effective in post‐procedure pain control and reducing opioid consumption.

Previous studies [11, 26] have consistently reported the advantages of using analgesic patches, emphasizing their association with fewer side effects. This is similar to our study that minimal occurrences of skin redness or nausea were observed among participants. Moreover, considering that post‐RFA pain is localized at the puncture site, a local analgesic would be more effective than oral or systemic analgesics in pain control, thereby minimizing side effects related to analgesics.

We calculated the total dosages of equivalent rescue analgesics in two groups at intervals of 0–6, 6–10, 10–14, 14–18, and 18–24 h. During the 0–6 h period, higher doses of equivalent rescue analgesics were administered, encompassing both oral and intravenous forms. The results indicated no significant difference between the two groups during the 0–6 h interval. However, the total dosages of equivalent rescue analgesics in the LP group were lower than those in the PP group during the 14–18 and 18–24 h intervals (Table S1). Interestingly, the application of the lidocaine patch would result in a reduction of oral analgesic use in one out of every seven treated individuals, particularly those with the lower pain score. Therefore, our study suggests that lidocaine patches should be made a viable option for managing post‐RFA pain.

Regarding our dropout rate, it was at only 14.4%. The experimental group experienced a dropout rate of less than 5%, whereas the placebo group exhibited a rate of 24%. According to the principles of evidence‐based medicine, a cutoff of 80% is commonly employed to determine the “levels of evidence,” distinguishing between “high” and “low” quality randomized trials [27]. Consequently, our experimental research is in accordance with high‐quality standards. The predominant proportion of attrition occurred in the placebo group rather than in the patch group, suggesting that the use of the lidocaine patch may help improve patient retention. This phenomenon may be attributed to the pain‐reducing effects of the lidocaine patch on most individuals, encouraging a greater willingness among patients to remain involved in the study.

### 4.1 Limitation

Lidocaine patches can superficially address pain at the puncture site. However, their effect on pain originating from deeper layers of the skin, the muscle, the peritoneum, or the liver parenchyma is limited. Additionally, we were unable to differentiate between superficial and deep pain or predict which patients would benefit from the local application of the lidocaine patch. This study only included short‐term follow‐up in the hospital for one day of pain assessment. Future studies should include a longer period of pain assessment following RFA treatment with the application of the lidocaine patch.

## 5 Conclusion

The study revealed that lidocaine patches reduce the need for rescue analgesics and the equivalent cumulative dose of residual morphine in HCC patients post‐RFA treatment. Therefore, the lidocaine patch can be suggested for HCC patients experiencing post‐RFA pain.

## Author Contributions

**Chen‐Ju Chen:** conceptualization (lead), data curation (lead), formal analysis (equal), methodology (lead), writing – original draft (lead), writing – review and editing (lead). **Wei‐Ying Chen:** conceptualization (lead), data curation (supporting), formal analysis (equal), investigation (equal), methodology (equal), writing – original draft (lead). **Chung‐Ying Lin:** methodology (lead), writing – original draft (equal). **Yen‐Cheng Chiu:** data curation (supporting), resources (lead). **Ying‐Ju Chang:** conceptualization (lead), data curation (supporting), formal analysis (supporting), methodology (supporting), supervision (lead), writing – review and editing (lead).

## Funding

This study was supported by National Cheng Kung University Hospital (NCKUH‐10707015).

## Disclosure

The authors have nothing to report.

## Ethics Statement

This study obtained approval from the Institutional Review Board of the National Cheng Kung University Hospital (B‐ER‐106‐060).

## Consent

Informed consent was obtained from all participants included in the study.

## Conflicts of Interest

The authors declare no conflicts of interest.

## Supporting information


**Table S1:** cam471581‐sup‐0001‐TableS1.docx.

## Data Availability

The data supporting this study's findings are available upon request from the corresponding author, Ying‐Ju Chang, upon reasonable request.
