# Correction to “The Effects of Lidocaine Patches on Post‐Radiofrequency Ablation Pain in Patients With Hepatocellular Carcinoma”

**DOI:** 10.1002/cam4.72200

**Published:** 2026-08-20

C.‐J. Chen , W.‐Y. Chen , C.‐Y. Lin , Y.‐C. Chiu , and Y.‐J. Chang , “The Effects of Lidocaine Patches on Post‐Radiofrequency Ablation Pain in Patients With Hepatocellular Carcinoma,” Cancer Medicine 15, no. 2 (2026): e71581, 10.1002/cam4.71581.41692439 PMC12906974

In the Methods section, under 2.1 Study Design, the sentence:

“This study was conducted with a block‐randomized clinical trial from December 2016 to December 2017 in a medical center of southern Taiwan” was incorrect.

This should read as follows:

“This study was conducted with a block‐randomized clinical trial from May 9, 2017 to April 30, 2018 in a medical center of southern Taiwan.”

We apologize for this error.

