# Supplementary material for: The Effects of Lidocaine Patches on Post‐Radiofrequency Ablation Pain in Patients With Hepatocellular Carcinoma
Source: Cancer Med. 2026 Feb 15;15(2):e71581. doi: 10.1002/cam4.71581 (PMC12906974; doi:10.1002/cam4.71581)
Supplement: Supplementary file 1 — Table S1: cam471581‐sup‐0001‐TableS1.docx. [file CAM4-15-e71581-s001.docx]

Supplement Table 1: The total equivalent dose of rescue morphine (mg) use in LP and PP groups, stratified by PO or IV and five time periods

| Time point | LP group (n=86) | | PP group (n=68) | |
| --- | --- | --- | --- | --- |
| Route | PO | IV | PO | IV |
| 0-6 hour | 112.5 | 136 | 187.5 | 155 |
| 6-10 hour | 37.5 | 0 | 0 | 0 |
| 10-14 hour | 0 | 0 | 0 | 20 |
| 14-18 hour | 0 | 18 | 117.5 | 0 |
| 18-24 hour | 0 | 0 | 155 | 10 |
